# Subculturing and Gram staining of blood cultures flagged negative by the BACTEC™ FX system: Optimizing the workflow for detection of *Cryptococcus neoformans* in clinical specimens

**DOI:** 10.3389/fmicb.2023.1113817

**Published:** 2023-03-15

**Authors:** Lingli Liu, Lijun Du, Shuquan He, Tianshu Sun, Fanrong Kong, Yali Liu, Yingchun Xu

**Affiliations:** ^1^Department of Laboratory Medicine, State Key Laboratory of Complex Severe and Rare Diseases, Peking Union Medical College Hospital, Chinese Academy of Medical Science and Peking Union Medical College, Beijing, China; ^2^Beijing Key Laboratory for Mechanisms Research and Precision Diagnosis of Invasive Fungal Diseases, Beijing, China; ^3^Department of Clinical Laboratory, Nanchong Central Hospital, the Second Clinical Medical College, North Sichuan Medical College, Nanchong, China; ^4^Jinan University, Guangzhou, Guangdong, China; ^5^Department of Clinical Laboratory, Longhua District Central Hospital, Shenzhen, China; ^6^Teaching Hospital of Guangdong Medical University, Guangdong, China; ^7^Medical Research Center, State Key Laboratory of Complex Severe and Rare Diseases, Peking Union Medical College Hospital, Chinese Academy of Medical Sciences, Beijing, China; ^8^Center for Infectious Diseases and Microbiology Laboratory Services, ICPMR—Pathology West, Westmead Hospital, University of Sydney, Westmead, NSW, Australia; ^9^Graduate School, Chinese Academy of Medical Sciences and Peking Union Medical College, Beijing, China

**Keywords:** bloodstream infection, subculture, blood culture, BACTEC™ FX, *Cryptococcus neoformans*

## Abstract

**Objective:**

To investigate whether an incubation time of 5 days (Aerobic/F, Anaerobic/F) and 14 days (Myco/F) blood culture bottles is sufficient to prevent false-negative results.

**Methods:**

We evaluated 1,244 blood bottles (344 patients) defined as negative by the BACTEC™ FX system. We also reviewed published cases and our own cases of bloodstream infection caused by *Cryptococcus neoformans* and simulated different scenarios, including different inoculation concentrations, bottle types, and clinical isolates.

**Results:**

Two bottles (0.16%) were found to contain *C. neoformans* when subcultured and Gram stained. A 5-day protocol with Aerobic/F bottles was insufficient for the growth of *C. neoformans* in some cases, and *C. neoformans* grew better in Myco/F bottles than in Aerobic/F bottles.

**Conclusion:**

Subculturing and Gram staining after a 5-day protocol were important for the detection of *C. neoformans*, and Myco/F bottles should be collected for the blood culture of *C. neoformans*.

## 1. Introduction

Bloodstream infection (BSI) is a major health burden worldwide, with mortality rates ranging from 20 to 50% (Magadia and Weinstein, 2001). Blood is one of the most important specimens received by the microbiology laboratory, as it plays an invaluable role in the detection and treatment of bacteremia and fungemia. Positive blood cultures should be promptly reported to clinicians, including the time-to-positivity, bottle type, Gram staining result, or the result of direct identification (Lamy, 2019). Some studies have confirmed that blood cultures incubated for <5 days are sufficient for the detection of infective endocarditis (Peuchant et al., 2019), neonatal sepsis (Biondi et al., 2014; Abdelhamid, 2017), or drug-resistant bacteria (Pan et al., 2019). However, most laboratories set the incubation time to 5–7 days (Janapatla et al., 2010; Ning et al., 2016; Cilloniz et al., 2017; Le Guern et al., 2021; Yarbrough et al., 2021) because the time-to-positivity of blood cultures can be influenced by blood volume in culture bottles (Chang et al., 2015; Kim et al., 2015; Jones et al., 2017; Birkhamshaw and Winzor, 2019; Henning et al., 2019; Khare et al., 2020), the initial inoculum (Klaerner et al., 2000), or prior exposure to antimicrobial agents (Driscoll et al., 2017). Like most laboratories, our laboratory sets the incubation time to 5 days with Aerobic/F bottles (14 days for yeast with Myco/F bottles, and 30 days for fungi with Myco/F bottles). We issue the first negative report at day 3 (Aerobic/F and Anaerobic/F bottles) and day 7 (Myco/F bottle), with the final report issued at day 5 and 14, respectively. Few studies have reassessed bottles defined as negative by Gram staining and/or inoculation on solid growth media (Peretz et al., 2015; Jacobs et al., 2017).

In our study, we evaluated 1,244 bottles defined as negative by the BACTEC™ FX system at the end of 5 days or 14 days (yeast) by using subculture and Gram stain. We also reviewed prior cases of BSI caused by *C. neoformans* and simulated several *in vitro* scenarios.

## 2. Materials and methods

### 2.1. Study design

The study was performed from September to October 2021 at Peking Union Medical College Hospital. Blood cultures (BACTEC™ PLUS-Aerobic/F Medium, BACTEC™-Lytic/10 Anaerobic/F Medium, and BACTEC™-Myco/F Lytic Medium; BD Diagnostics, Oxford Science Park, Oxford, United Kingdom) were sent to the microbiology laboratory. Distribution of patients was across different departments including emergency, intensive care unit, respiratory disease, cardiology, immunology, infectious diseases, neurology, and oncology. Aerobic/F and Anaerobic/F bottles were incubated in the BACTEC™ FX system for 5 days, and Myco/F bottles were incubated for 14 days for yeasts, such as *Candida*. The acceptable time period from bottle collection to placement onto the automated blood culture instruments should be within 2 h, and all of the bottles detected in the present study fulfilled this criteria.

### 2.2. Subculturing and Gram staining of negative bottles

**Figure 1:**
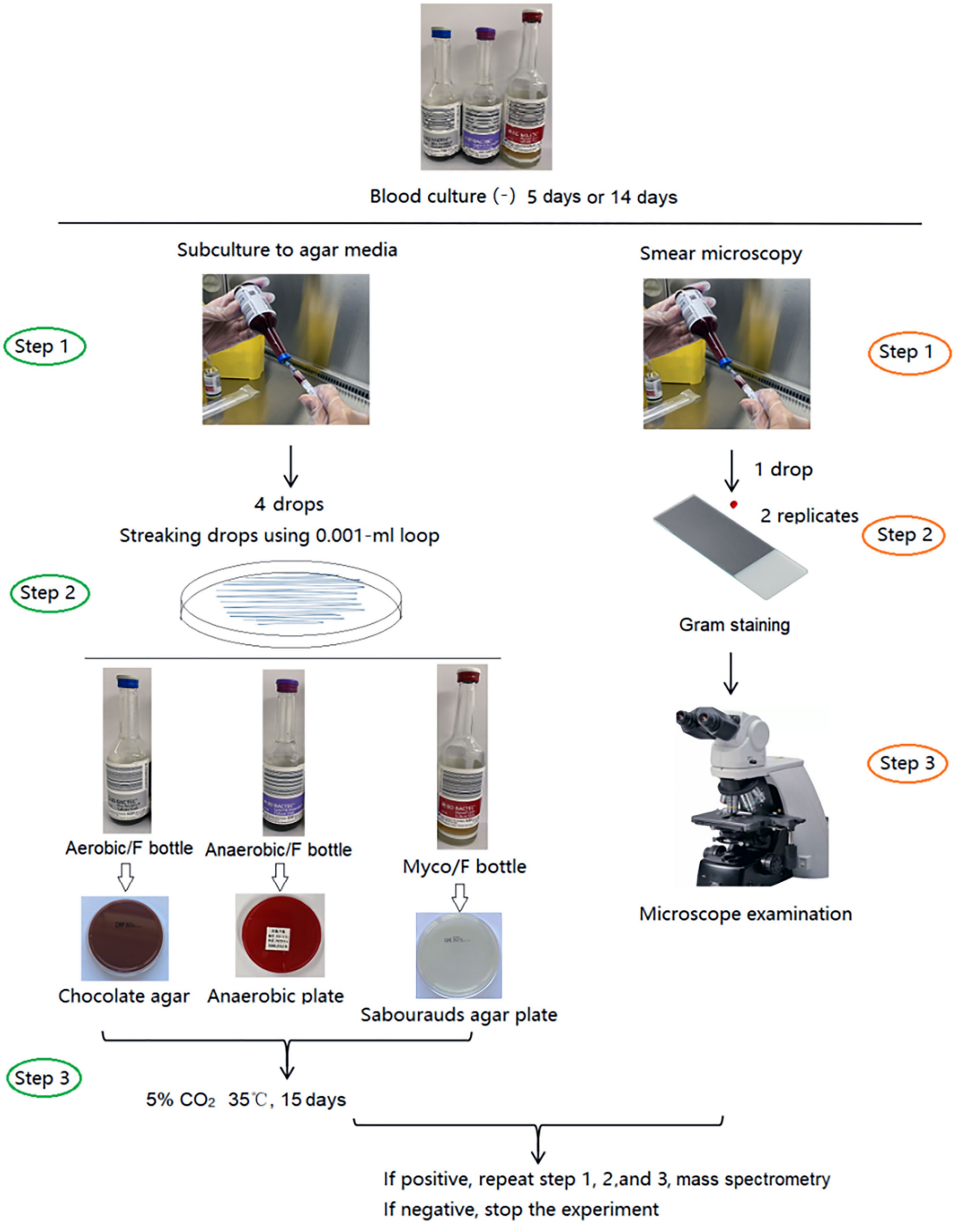
Study design.

Blood bottles, defined as negative by the BACTEC™ FX system after 5 days (Aerobic/F and Anaerobic/F bottles) or 14 days (Myco/F bottle), were subcultured and Gram stained (smear microscopy). The Aerobic/F bottles were subcultured onto chocolate agar, the Anaerobic/F bottles were subcultured onto anaerobic blood agar (Schaedler agar base supplemented with 5% sheep blood, vitamin K1, and hemin) and sealed in a disposable anaerobic bag (GENbag, Chemin de I’Ome 69280 Marcy L’ETOILE, France), and the Myco/F bottles were inoculated onto Sabouraud’s agar. To improve the subculture yield, the inoculum (four drops) was streaked closely and evenly, and cultured in 5% CO_2_ at 35°C for 15 days. Subcultured plates were reviewed for growth on days 2, 7, and 15 after inoculation according to our study design. If colonies were observed, they were identified by mass spectrometry (Antubio, China) and the original blood culture bottles were subcultured and Gram stained again to exclude possible contaminants (Figure 1). Contaminants or true pathogens were determined according to the criteria listed in Table 1.

**Table 1:** Criteria used to assess contaminants or causative pathogens.

Subculturing	Gram staining	Clinical diagnosis supporting BSI	Contaminants or Causative pathogens
+ (confirmed twice)	+ (confirmed twice)	Yes	Causative pathogens
+ (confirmed twice)	− (confirmed twice)	Yes	Causative pathogens
− (confirmed twice)	+ (confirmed twice)	Yes	Causative pathogens^*^
+1st/−2nd or + (confirmed twice)	+ 1st/−2nd or + (confirmed twice)	No	Contaminants

* Metagenomic next-generation sequencing was used for the identification of pathogens. BSI, bloodstream infection.

When the original blood culture bottles were negative on subculturing but positive by Gram staining, metagenomic next-generation sequencing (Illumina NextSeq CN500) would be used for the identification of pathogens. The concentration of extracted DNA/RNA was measured using a Qubit Fluorometer before library preparation. DNA libraries were prepared *via* transposase-based methodology. Human rRNA was depleted from the RNA samples *via* an RNase H-based method before library preparation. After purification and size selection, the concentration of the RNA library was determined by using a Qubit instrument before pooling. Pooled libraries were sequenced on an Illumina NextSeq 550 system using a 75 bp, single-end sequencing kit (Illumina, San Diego). The qualified results had no fewer than 15 million reads obtained per sample and a Q30 score of 90% or greater. A negative control sample was processed and sequenced in parallel in each sequencing run for quality control. The 75 bp single-end reads from illumine Nextseq 550 were analyzed by in-house IDseq software to get each microorganism’s abundance. The detail process is as follows: high-quality sequencing data were generated by removing reads of low quality or short length (<35 bp) by using fastp (Chen et al., 2018). Human host sequences were subtracted by mapping to human reference genome sequences (National Center for Biotechnology Information GRCh38 assembly) using the Burrows-Wheeler Aligner tool (BWA) 1 (Li and Durbin, 2010). The data remaining after the removal of low-complexity reads were classified by alignment to curated microbial genome databases for viruses, bacteria, fungi, and parasites. Taxonomic references were downloaded from the National Center for Biotechnology Information.

### 2.3. Evaluation of different bottle types, sample volumes, and clinical isolates

**Figure 2:**
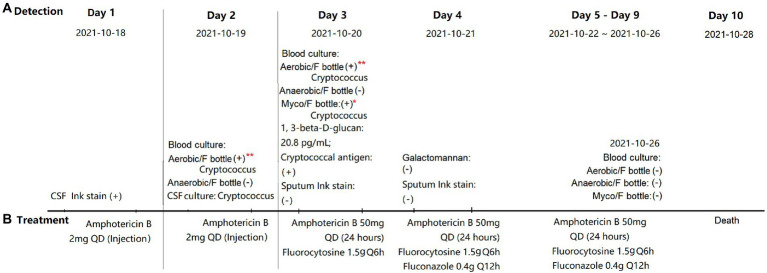
Detection **(A)** and treatment **(B)** of *Cryptococcus neoformans* infection. ^*^Defined as positive by the BACTEC™ FX system at 82 h; ^**^defined as negative by the BACTEC™ FX system at the end of 5 days but *C. neoformans* was detected on subculturing and yeast-like cells were observed by Gram staining. ER, emergency room; ICU, intensive care unit; CSF, cerebrospinal fluid; QD, means once a day. Q6h, means take medicine every 6 h; and Q12h, means take medicine every 12 h.

Four *C. neoformans* isolates (Figure 2), namely H99 (*C. neoformans* reference clinical strain), 21B29228 (isolated from a patient in the present study; October 19, 2021), 21B29353 (isolated from the same patient; October 20, 2021), and 21B29354 (isolated from the same patient; October 20, 2021), were each serially diluted to final concentrations of 5–10, 50–100, and 500–1,000 colony-forming units (CFU)/ml in sterile saline. To simulate the ratio of 1:5/1:10 (blood volume/media), Aerobic/F bottles were inoculated with 10 mL of the three concentrations, giving final inoculation concentrations of 50–100, 500–1,000, and 5,000–10,000 CFU/bottle, respectively. Myco/F bottles were inoculated with 5 and 10 mL of the three concentrations to final inoculation concentrations of 25/50–50/100, 250/500–500/1,000 and 2,500/5,000–5,000/10,000 CFU/bottle, respectively. To enumerate the exact CFU inoculated, 100 μL of each concentration was plated on 5% Sabouraud’s agar plates, and colonies were counted after 48 h incubation at 35°C. Bottles were cultured in the BACTEC™ FX system until they appeared positive or until 7 days. The time-to-detection (TTD; defined as the time from loading to observation of a positive signal in the blood culture) was determined for each bottle. Bottles that failed to show a positive result were subcultured on Sabouraud’s agar plates after being unloaded.

### 2.4. Statistical analysis

**Figure 3:**
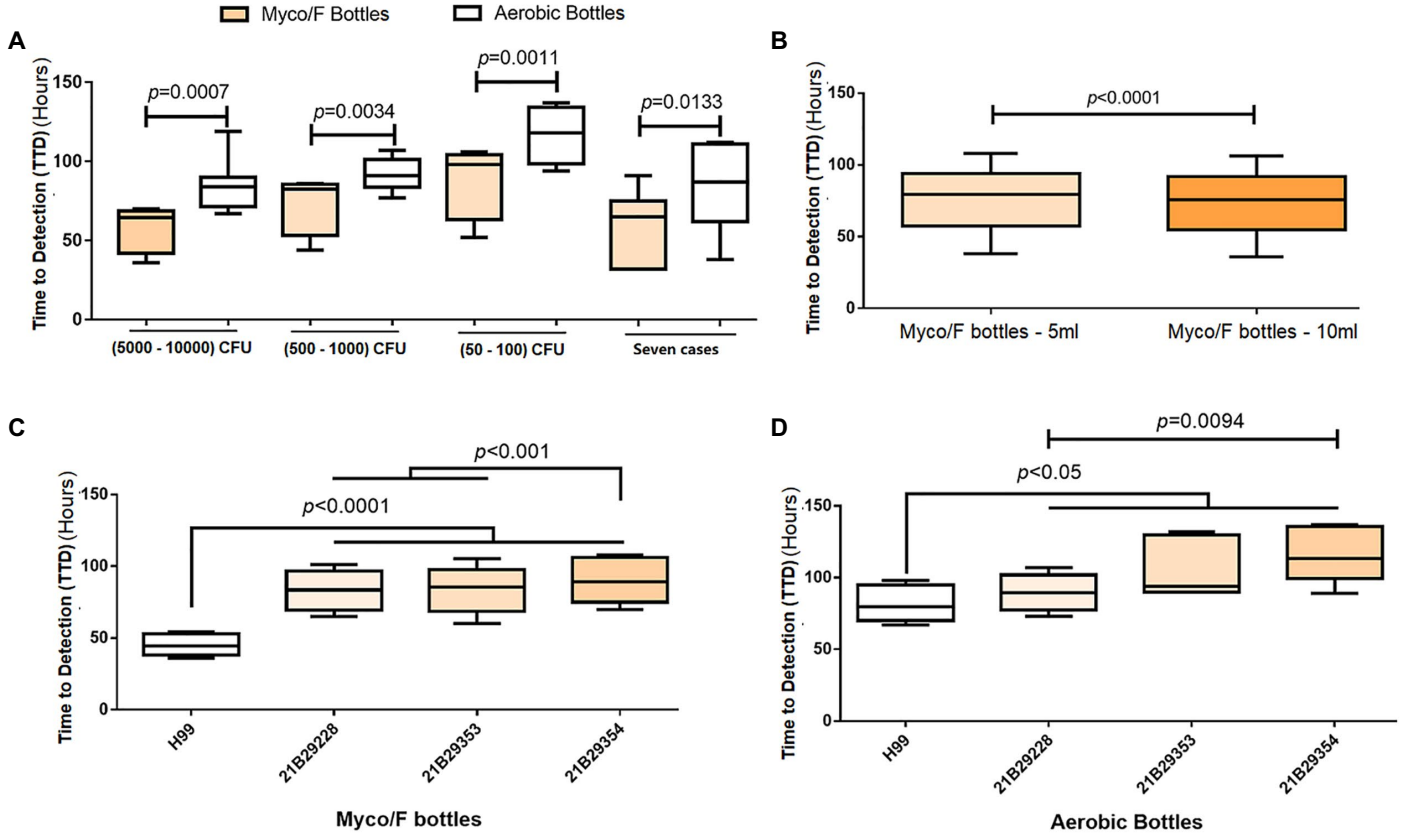
Evaluation of different blood bottles, sample volumes, and clinical isolates. **(A)** Comparison of the time-to-detection (TTD) between Aerobic/F and Myco/F bottles as well as various inoculation concentrations. Eight replicates for each inoculation concentration. **(B)** Comparison of the TTD between 5-and 10-mL samples in Myco/F bottles. Twelve replicates for each volume (5- or 10-mL). **(C)** Comparison of the TTD among four clinical *C. neoformans* isolates inoculated in Myco/F bottles. Twelve replicates for each isolate. **(D)** Comparison of the TTD among four clinical *C. neoformans* isolates inoculated in Aerobic/F bottles. Six replicates for each isolate.

Differences in the TTD between Myco/F and Aerobic/F bottles (Figure 3A) or between 5-and 10-mL samples (Figure 3B) were evaluated using a paired *t* test (two-tailed). Differences in the TTD among four different isolates (Figures 3C,D) were compared using one-way ANOVA (multiple comparisons). All statistical analyses were performed using GraphPad Prism, version 7.0, and differences were considered statistically significant at *p* < 0.05.

## 3. Results

We collected, subcultured, and Gram-stained 542 Aerobic/F bottles, 544 Anaerobic/F bottles, and 158 Myco/F bottles (all from 344 patients) defined as negative. Among the 542 Aerobic/F bottles, bacteria or fungi grew in four of the subcultured bottles and yeast-like cells (*C. neoformans* yeast cells were very round and displayed an amorphous orange-staining material, presumably the capsule) were detected in two of the Gram-stained bottles. In the two bottles that tested positive by subculturing and gave a negative smear result, *Saccharomyces cerevisiae* was detected in one bottle and *Bacillus* sp. in the other bottle. However, both tested negative after a second round of subculturing and Gram staining, and these organisms were therefore considered to be contaminants. For two of the subcultured bottles, which were collected from the same patient (21B29228, October 19, 2021; and 21B29354, October 20, 2021), *C. neoformans* was identified by mass spectrometry and yeast-like cells were also observed by Gram staining (Figure 2). The blood culture in the Myco/F bottle collected from the same patient was reported positive at 82 h (21B29353, October 20, 2021; Figure 2) and identified as *C. neoformans* by mass spectrometry. The subcultured and Gram-stained 544 Anaerobic/F bottles and 158 Myco/F bottles were negative. Therefore, the overall positive rate for re-examination of 1,244 bottles defined as negative according to our protocol was 0.16% (2/1244).

We reviewed the past 7 years of our laboratory records (unpublished data) and found 17 cases of BSI caused by *C. neoformans.* Among the 17 cases, three (17.6%) were detected only in Myco/F bottles; seven (41.2%) were detected in both Myco/F and Aerobic/F bottles; two (11.8%; 72 and 153 h) were detected only in Aerobic/F bottles; and five (29.2%) were cultured solely in Aerobic/F cultures and reported positive. For the 10 cases (58.8%) detected in Myco/F bottles, the average TTD was 75.4 h, and nine isolates of *C. neoformans* grew within the recommended incubation time (7 days) for yeast using Myco/F bottles (one required 179 h for growth). We compared the TTD between Myco/F and Aerobic/F bottles in seven cases (Figure 3A). The average ± SD TTD for Myco/F bottles was 60.57 ± 21.72 h, and that for Aerobic/F bottles was 84.00 ± 27.26 h, and the difference was significant (*p* = 0.0133).

We recovered the three *C. neoformans* isolates detected in the present study, namely 21B29228, 21B29353, and 21B29354, and H99 was used as a growth control. We seeded the four isolates into Myco/F and Aerobic/F bottles. *Cryptococcus neoformans* isolates grew faster in the Myco/F bottles than in the Aerobic/F bottles (Figure 3A), with time differences of 26.5, 18.3, and 28.3 h at inoculation concentrations of 5,000–10,000, 500–1,000, and 50–100 CFU/bottle, respectively (*p* < 0.05). A high blood volume (indicating a high inoculum) shortened the average TTD from 77.00 ± 22.07 to 73.42 ± 21.77 h (*p* < 0.05; Figure 3B). Isolates 21B29228, 21B29353, and 21B29354 grew slower than H99 (Figure 3C), and 21B29354 grew slower than 21B29353 and 21B29228 (Figure 3D; *p* < 0.05).

## 4. Discussion

Because bacteremia and fungemia are life-threatening infections, the detection and identification of microorganisms from blood are crucial. Blood culture is commonly based on the measurement of CO_2_ production, such as with the BACTEC™ FX (BD Diagnostics) system (Chang et al., 2015; Peretz et al., 2015), or the measurement of CO_2_-derived pH changes, such as with the BacT/ALERT system (bioMérieux; Kim et al., 2015; Jacobs et al., 2017; Emeraud et al., 2021), by which 99% of positive Aerobic/F and Anaerobic/F bottles with BacT/Alert Virtuo (Virtuo) blood culture detection system are confirmed to be positive within 5 days (Ransom et al., 2021). However, false-negative results can arise when the incubation time is set to 5 or 7 days, because of slowly proliferating microorganisms (Peretz et al., 2015), insufficient blood volume (Kim et al., 2015), or prior use of antimicrobial agents (Houpikian and Raoult, 2005).

To improve detection procedures, the current study reviewed blood cultures that were reported as negative according to our hospital’s protocol. Two Aerobic/F bottles tested positive for *C. neoformans* (Figure 2). As a Myco/F bottle for this patient was flagged as positive at 82 h (within the recommended 7 days incubation for Myco/F culture of yeast), these false-negative bottles would not have affected the clinical management. However, our blood culture audit did reveal occasions when the diagnosis of cryptococcemia relied solely on the Aerobic/F bottles. Thus, it is important to explore the potential factors contributing to the failure of the Aerobic/F bottles to detect *Cryptococcus*. We hypothesize that the presence of antifungals, a short incubation time, and a small blood volume may contribute.

Initial treatment with amphotericin B was implemented immediately following primary diagnosis of cryptococcal meningitis using cerebrospinal fluid ink staining. Blood was collected before the next dose was administered (24 h; Figure 2), so growth inhibition by amphotericin B could not be predicted (immediate treatment was needed, which can obscure subsequent testing results). We recovered three isolates (21B29354, 21B29353, and 21B29228) and reseeded them into Myco/F and Aerobic/F bottles (at concentrations of 5,000–10,000, 500–1,000, and 50–100 CFU/bottle) in the absence of any antifungal agents. Myco/F bottles reseeded with 21B29354, 21B29353, and 21B29228 flagged positive at 82 h, at a concentration of 500–1,000 CFU/bottle (data not shown here). At that concentration, all three isolates reseeded into Aerobic/F bottles flagged positive at 89–107 h, within the recommended incubation time of 5 days. However, at a concentration of 50–100 CFU/bottle, isolates 21B29354 and 21B29353 flagged positive at 129–137 h, outside the recommended incubation time of 5 days. If the lack of detection of Aerobic/F bottles is attributable to antifungal agents, the shorter TTD in Myco/F bottles cannot be explained because Aerobic/F bottles contain resin that can adsorb antimicrobial agents, but Myco/F bottles do not. However, there are two possible explanations: (1) the media in Myco/F bottles facilitates better growth of *C. neoformans* compared with that in Aerobic/F bottles (which remains to be confirmed) and (2) the lower initial inoculum in Aerobic/F bottles prolongs the TTD (as confirmed by the results mentioned above).

Previous studies using Difco ESP 384 (Robinson et al., 1987; Tinghitella and Lamagdeleine, 1995), BACTEC™ (Araj et al., 1981), and BBL Microbiology (Gill, 1981; Table 2) systems also reported that Aerobic/F bottles failed to detect *C. neoformans* isolates with a 5- or 7-day protocol (Table 2). To evaluate the comparative TTD of Myco/F and Aerobic/F bottles for *C. neoformans*, we reviewed the past 7 years of data. We collected 17 BSI cases caused by *C. neoformans* from our hospital (unpublished data), and compared the TTD reported for Myco/F and Aerobic/F bottles (Figure 3A). Myco/F bottles detected *C. neoformans* 23.43 ± 17.88 h prior to Aerobic/F bottles, based on the BACTEC™ FX system. Experiments using the four recovered *C. neoformans* isolates gave similar results, with *C. neoformans* being detected in Myco/F bottles 24.33 ± 13.52 h prior to Aerobic/F bottles, based on the BACTEC™ FX system (Figure 3A). Furthermore, in five previous cases of BSI caused by *C. neoformans*, Myco/F bottles were not designated for use in the whole process. Indeed, most of our *C. neoformans* isolates in Aerobic/F and Myco/F bottles were detected within 5 days, but some isolates needed a longer time (one case flagged positive at 153 h in an Aerobic/F bottle, and another at 179 h in an Myco/F bottle). This may have resulted from phenotypic differences among the strains or the initial inoculum concentration in the blood. Growth of the three *C. neoformans* isolates in the present study was slower than the growth of control H99 and other isolates reported previously (within 72 h; Zhang et al., 2019). Similarly, 21B29354 collected on October 20, 2021 grew slower than 21B29228 collected on October 19, 2021 (*p* < 0.05), despite being derived from the same patient. The growth curves of the three bottles were checked. An extended incubation time for the two Aerobic/F bottles was required because they were likely to flag positive after 5 days. Therefore, a 5-day protocol with Aerobic/F bottles was insufficient for the growth of *C. neoformans* in some cases, and Myco/F bottles performed better than Aerobic/F bottles for the growth of *C. neoformans.*

**Table 2:** Literature review of automated blood culture systems that failed to detect *Cryptococcus neoformans.*

Blood culture system	Detection methods	Incubation time in automated blood culture system	No. of subcultured positive cases	Microbe detected in negative blood culture bottles	References
Difco ESP 384	Gram staining and Subcultivation	5 days	16 (1.4%; 16/1162)	*Cryptococcus neoformans* (*n* = 8)*, Candida albicans* (*n* = 1)*, Staphylococcus aureus* (*n* = 2)*, Coagulase-negative staphylococcus* (*n* = 3), *Bacillus* sp. (*n* = 1), *and Corynebacterium sp.* (*n* = 1)	Tinghitella and Lamagdeleine (1995)
Bactec® 460 Radiometric	Subcultivation	7 days	Eight (in a patient with AIDS)	*Cryptococcus neoformans* (*n* = 8)	Robinson et al. (1987)
BACTEC	Subcultivation	7 days	15 (0.3%; 15/5345)	*Cryptococcus neoformans* (*n* = 8), *Staphylococcus epidermidis* (*n* = 1), *Bacillus cereus* (*n* = 1) *Corynebacterium sp.* (*n* = 1) *Coccidioides immitis* (*n* = 4)	Araj et al. (1981)
	Subcultivation	7 days	12 (0.8%; 12/14,000)	*Cryptococcus neoformans* (*n = 1*), *Staphylococcus aureus* (*n* = 2), *Pseudomonas aeruginosa* (*n* = 2), *Candida tropicalis* (*n* = 3), *Staphylococcus epidermidis* (*n* = 1), *Neisseria meningitidis* (*n* = 1), *Acinetobacter calcoaceticus subsp. anitratus* (*n* = 1), and *Klebsiella pneumoniae* (*n* = 1)	29
BACTEC FX	Gram staining and subcultivation	5 days	2 (1.6‰; 2/1244)	*Cryptococcus neoformans* (*n* = 2)	Present study

According to the manufacturer’s instructions, Myco/F bottles should be seeded with up to 5 mL of blood. We compared the TTD between the 5- and 10-mL samples and confirmed that the latter shortened the average TTD by 3.59 ± 2.83 h (*p* < 0.05). Therefore, we suggest that the high blood volume or high inoculum concentration in Myco/F bottles may have contributed to the positive results.

We also compared the TTD between Aerobic/F bottles with 10-mL samples and Myco/F bottles with 5-mL samples at three concentrations. Myco/F bottles detected *C. neoformans* on the BACTEC™ FX system 25.75 ± 14.13 h (5,000–10,000 CFU/bottle), 14.75 ± 12.40 h (500–1,000 CFU/bottle), and 21.75 ± 13.17 h (50–100 CFU/bottle) prior to Aerobic/F bottles, which also confirmed the preferability of Myco/F bottle use for the detection of *C. neoformans.*

We also reviewed the time from sampling to loading of the bottles in the incubation system and confirmed that all bottles collected from this patient infected by *C. neoformans* were loaded into the BACTEC™ FX system within 1 h of blood culture collection. Thus, the difference in the ability to recover *Cryptococcus* was unlikely caused by transport and/or a delay in loading.

On the basis of the results above, we were able to optimize our workflow in four parts. (1) When a clinical diagnosis of cryptococcal infection is made or suspected, Myco/F bottles should be collected, and the incubation time for Aerobic/F bottles should be extended to 14 days. If the Aerobic/F bottles are defined as negative at 5 days, they should be subcultured and Gram stained. (2) When a positive result is obtained for ink staining of the cerebrospinal fluid and/or sputum, and/or cryptococcal antigen is positively detected, Myco/F bottles should be collected, and the incubation time of Aerobic/F bottles should be extended to 14 days. If the Aerobic/F bottles are defined as negative at 5 days, they should be subcultured and Gram stained. (3) When one or two bottles of a set of bottle cultures (Aerobic/F bottles) are flagged positive, the incubation time for other bottles (Aerobic/F bottles) from the same set and other sets should be extended to 14 days and they should be subcultured and Gram stained at 5 days (Aerobic/F bottles). (4) To increase the pre-test probability of a patient having *C. neoformans* infection (e.g., known exposure, immunosuppression due to HIV, chemotherapy, etc.), subculture and Gram staining of the bottles at the end of routine incubation times would also be performed.

In addition to optimizing our workflow, future studies are necessary to assess more rapid and sensitive approaches (Iroh Tam et al., 2018; Zrodlowski et al., 2018; Kidd et al., 2019; Ogbebor et al., 2021; Pecoraro et al., 2021), such as quantitative real-time PCR, nested multiplex real-time PCR, next-generation sequencing, and fluorescent *in situ* hybridization. Human blood has traditionally been considered to be an entirely sterile environment, but evidence for the existence of a healthy human blood microbiome is steadily accumulating (Castillo et al., 2019). When deciding whether microorganisms are contaminants or causative pathogens, results should be interpreted according to the clinical context. In the cases reported in this study, *C. neoformans* was definitely a causative pathogen.

Our study had some limitations. (1) We only collected a small number of bottles defined as negative by the BACTEC™ FX system, and more relevant cases may be found if a larger number of bottles were included. (2) We analyzed and calculated results according to bottles, not patients (*n* = 344); therefore, some bottles were collected from the same patients. (3) We did not evaluate the BacT/ALERT system because of the small number of bottles used in our hospital. (4) This study made the organism dilutions using saline not human blood, which may skew the results toward prolonged organism recovery/growth in the blood culture bottles. (5) Due to different growth rates between the patient isolate and the control isolate, the observed TTD data may not be generalized globally. And the modified workflow is a proof of concept study, which needs to be further validated using many different strains of *C. neoformans* from different parts of the world.

Overall, we found that a 5-day protocol with Aerobic/F bottles was insufficient for the growth of *C. neoformans* in some cases. Furthermore, Myco/F bottles performed better than Aerobic/F bottles regarding the growth of *C. neoformans.* Subculturing and Gram staining after a 5-day protocol were important for the detection of *C. neoformans*, and laboratories should ensure that Myco/F bottles are used for blood culture of *C. neoformans*.

## Data availability statement

The raw data supporting the conclusions of this article will be made available by the authors, without undue reservation.

## Ethics statement

The studies involving human participants were reviewed and approved by only de-identified, clinically-obtained, bacterial isolates were used in the present study; no human subjects were involved (including the use of tissue samples). The study was approved by the Human Research Ethics Committee of Peking Union Medical College Hospital (No. ZS-3260). Written informed consent for participation was not required for this study in accordance with the national legislation and the institutional requirements.

## Author contributions

YL designed the study. YL, LL, and LD analyzed the data. LL, LD, and SH performed the experiments. LL, LD, and TS prepared the experimental materials. YL wrote the original manuscript. YL and FK revised the manuscript. YX supervised the project. All authors contributed to the article and approved the submitted version.

## Funding

This work was supported by the Special Foundation for National Science and Technology Basic Research Program of China (No. 2019FY101200), the National Key Research and Development Program of China (No. 2021YFC2400905), and the Beijing Key Clinical Specialty for Laboratory Medicine-Excellent Project (No. ZK201000).

## Conflict of interest

The authors declare that the research was conducted in the absence of any commercial or financial relationships that could be construed as a potential conflict of interest.

## Publisher’s note

All claims expressed in this article are solely those of the authors and do not necessarily represent those of their affiliated organizations, or those of the publisher, the editors and the reviewers. Any product that may be evaluated in this article, or claim that may be made by its manufacturer, is not guaranteed or endorsed by the publisher.
